# Joint line reestablishment in revision total knee arthroplasty

**DOI:** 10.1186/s42836-020-00046-4

**Published:** 2020-09-14

**Authors:** Mustafa Çınar Akça, Yavuz Akalın, Nazan Çevik, İsmail Gökhan Şahin, Özgür Avcı, Alpaslan Öztürk

**Affiliations:** 1 Research and Training Hospital Clinic of Orthopaedics and Traumatology, Health Sciences University Bursa YuksekIhtisas, 16310, Yildirim, Bursa, Turkey; 2 Clinic of Orthopaedics and Traumatology, Turkish Ministry of Health, Edirne Sultan 1 Murat State Hospital, Edirne, Turkey

**Keywords:** rTKA, Adductor tubercle, Tibial tubercle, Ratios method, Anatomical landmark, Distance method, Joint line (JL)

## Abstract

**Background:**

In this study, the traditional “Anatomical Landmark-Distance Method (AL-DM)” in the formation of joint line (JL) was compared with “Adductor Tubercle-Ratios method” (AT-RM), and the effect of reestablishment of JL on clinical and functional outcomes were evaluated.

**Materials and methods:**

16 revision total knee arthroplasties (rTKAs) were performed by using “AT-RM” (group 1) and 16 rTKA by using “AL-DM” (group 2) in our clinic between 2015 and 2018. The data were prospectively collected and a total of 32 knees of 31 patients were analyzed. At the final follow-up, knee functions were evaluated by using Knee Society Score (KSS) knee and function, Western Ontario and McMaster Universities Arthritis Index (WOMAC) scores, Short Form-36 (SF-36) questionnaires and physical examinations.

**Results:**

Postoperative flexion arc was higher in Group 1. KSS knee and function scores were better in group 1. In group1, JL was reestablished successfully in all revision rTKAs in terms of ATJL and the tibial tubercle TT-JL ratios. The improvement in KSS knee and function scores and WOMAC scores were also better in group 1. Measurements showed that the improvement in KSS scores increased as AT-JL and TT-JL distances approached the calculated values.

**Conclusion:**

“AT-RM” was shown to be superior to the traditional distance method in terms of JL reestablishment. Functional results and patient satisfaction increased when JL was reestablished.

## Introduction

Joint line (JL) restoration is a prerequisite for a successful revision total knee arthroplasty (rTKA) [1]. It has been reported that there was a19 millimeter (mm) JL elevation and a 10 mm depression, even with primary TKA [2]. JL elevation of more than 8 mm was reportedly associated with unfavorable clinical results [3]. Moreover, a recent study showed that elevation over 4 mm was related to lower patellofemoral function [4].

Anatomical structures around the knee, such as medial and lateral epicondyles (ME, LE), tibial tubercle (TT) and fibular head (FH) have been used for calculation and restoration of JL in rTKA [5–7]. However, precept distances like ‘2 cm proximal to the fibular head’ or ‘1.5 – 2 cm distal to the lateral epicondyle’ cause JL to be elevated by more than 5 and 8 mm in rTKA because there are obvious variations in terms of race, gender and body mass index [8–14]. Given all these disadvantages, some new techniques, such as methods of ratios of femoral width (FW) and adductor tubercle (AT), ME-JL, LE-JL and TT-JL have been proposed to reestablish the coronal plane JL and posterior JL (PJL) in rTKA and good results have been reported in recent years [1–3, 7, 9, 10, 12, 14]. Besides, the posterior JL is also known as the posterior femoral offset affecting the flexion gap and it has been reportedly established to be the poterior one-third of the clinical trans-epicondylar axis (TEA) [11].

In the current study, we aimed to compare the postoperative clinical and radiological results of rTKAs in terms of JL reestablished with ATRM and ALDM.

## Materials and methods

The study was conducted in our clinic between March 2015 and January 2018. 32 rTKAs were performed in 31 patients (1 received bilateral rTKA). Of them, 16 rTKAs were done with ‘Adductor Tubercle-Ratios Method (AT-RM)’ (group 1) and the remaining 16 rTKAs received the ‘Anatomic Landmark-Distances Method (AL-DM)’ (Group 2) method. Twenty-one patients were female and 10 were male.

Reason for revision included a periprosthetic joint infection in 9 (28.1%), instability in 2 (6.3%) and aseptic loosening in 21 (65.6%) knees. Twenty-three rTKAs (71.9%) were performed in one stage and the other 9 (28.1%) were done in two stages (Table 1).

**Table 1:** Etiological factors for rTKAs

	**Group 1**	**Group 2**	***p*** **value**^**a**^
**Gender**			
**Male / Female**	6 / 10	4 / 11^b^	0.704
**Reason**			
**Aseptic Loosening**	11^c^	10	0.010
**Periprosthetic Joint Infection**	4	4^b^	0.166
**Instabilty**	1	1	0.014
**Revision Stage**			
**One / Two**	12^c^/ 4	11 / 4^b^	1.000

^a^Chi-Square Test,^b^One bilateral rTKAs, ^c^Two patients died in group 1

Surgeries were carried out under combined spinal-epidural or general anesthesia. Tibial tubercle osteotomy was needed in 2 knees in group 1. Bone defects were classified according to the Anderson Orthopedic Research Institute (AORI) classification [15].

### Typical case in group 1

**Fig. 1:**
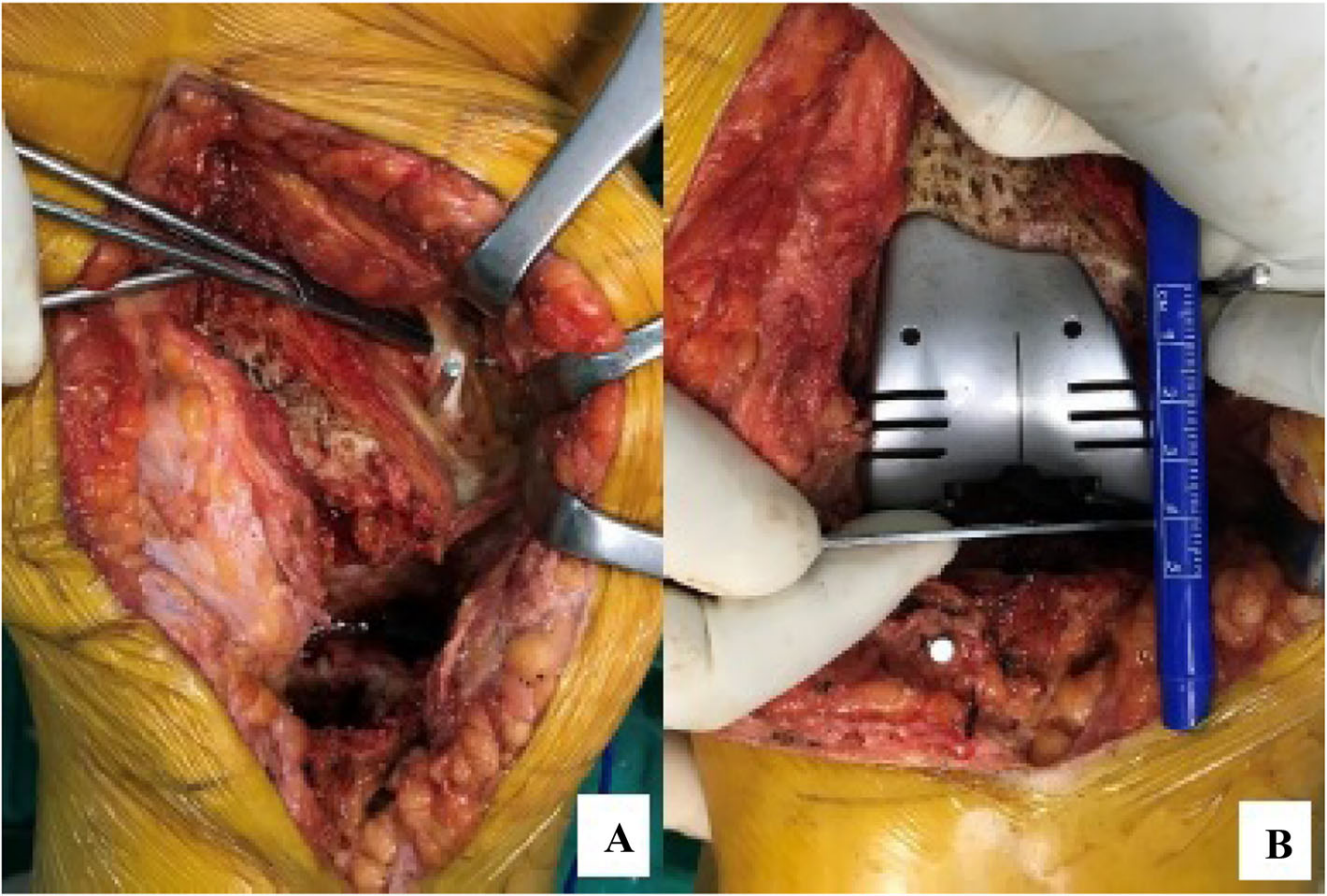
**a.** Kirshner wire placed at the insertion of the Adductor Magnus muscle (Adductor Tubercule). **b.** Joint line restorations with femoral trial prothesis according to calculations from AT

FW was measured with a ruler. The most common mistake in placing the femoral prosthesis lies in that it is placed more proximally and more anteriorly, and because no sufficient heed was paid to the posterior condylar offset (PCO), the femoral prosthesis used tends to be smaller than what it should be (12). In view of this, we used femoral offset in all cases. Then, as described by Servien et al (9), FW was multiplied by 0.53 to obtain the AT-JL distance. By estimating the size of the femoral prosthesis at this stage, the joint line was restored with femoral trial prothesis according to calculations from AT (Fig. 1).

**Fig. 2:**
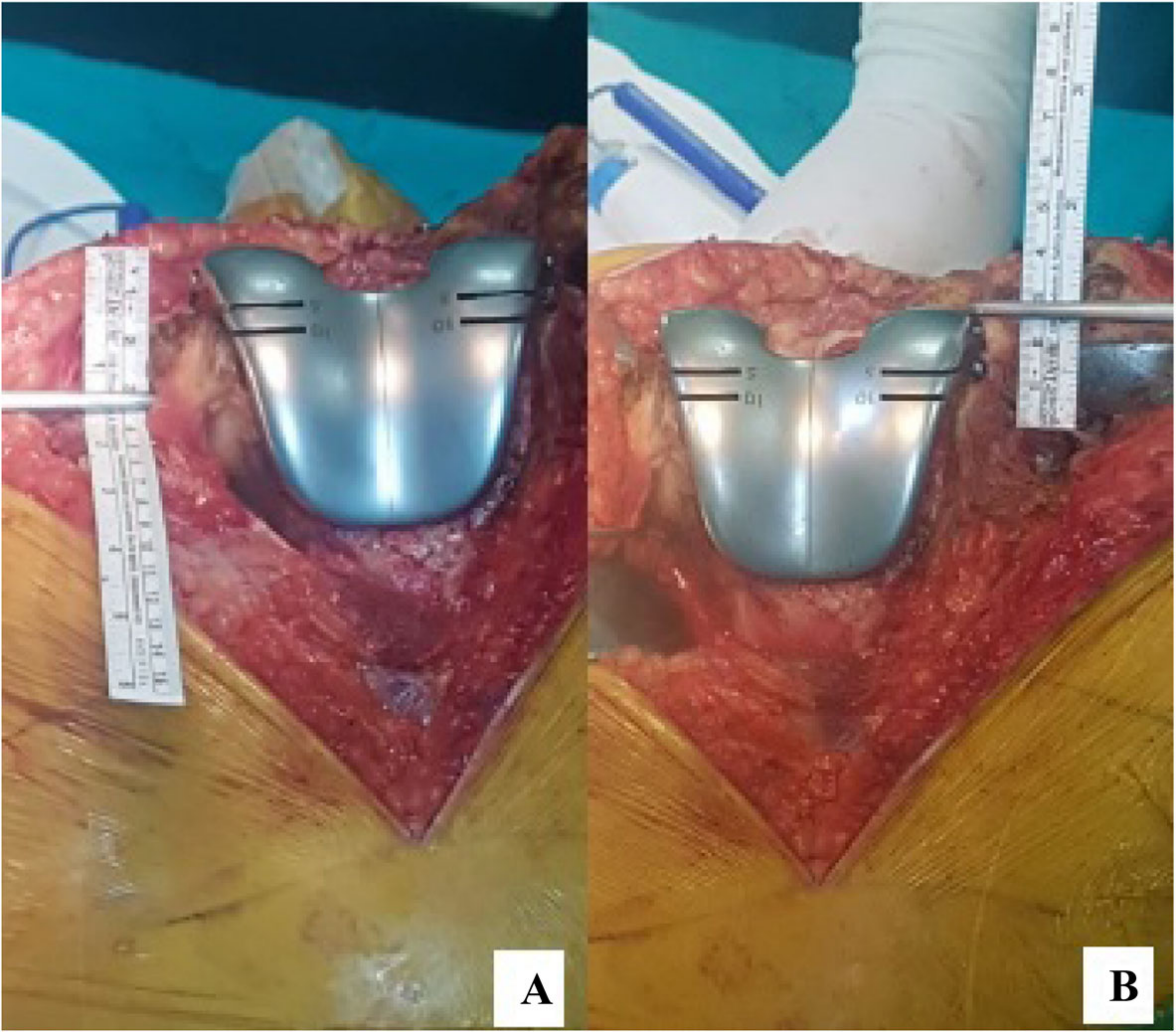
Joint line restoration with femoral femoral trial prothesis according to calculations. **a.** ME-JL. **b.** LE-JL

**Fig. 3:**
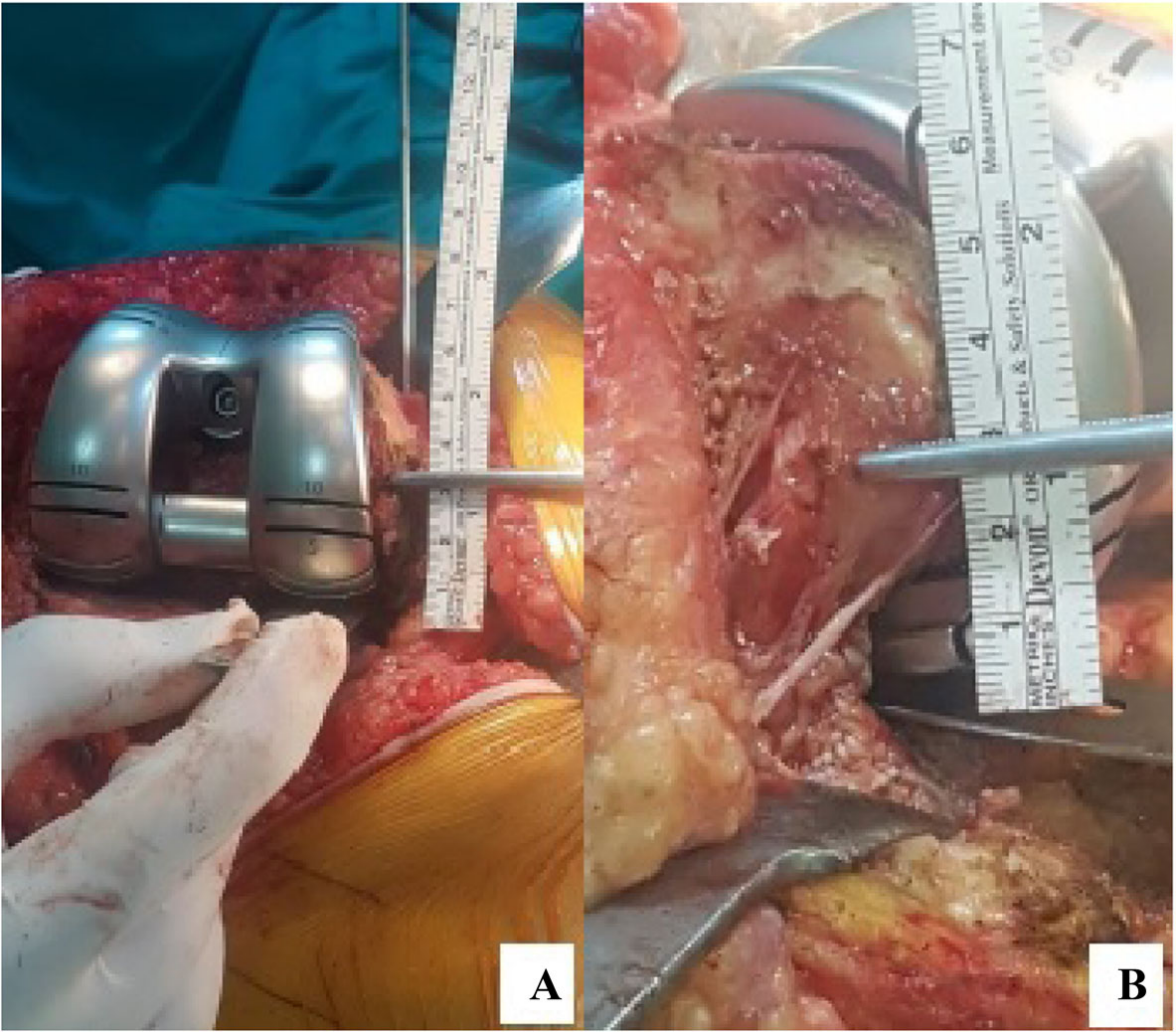
Posterior joint line restorations with femoral trial prothesis according to calculations. **a.** ME-PJL. **b.** LE-PJL

**Fig. 4:**
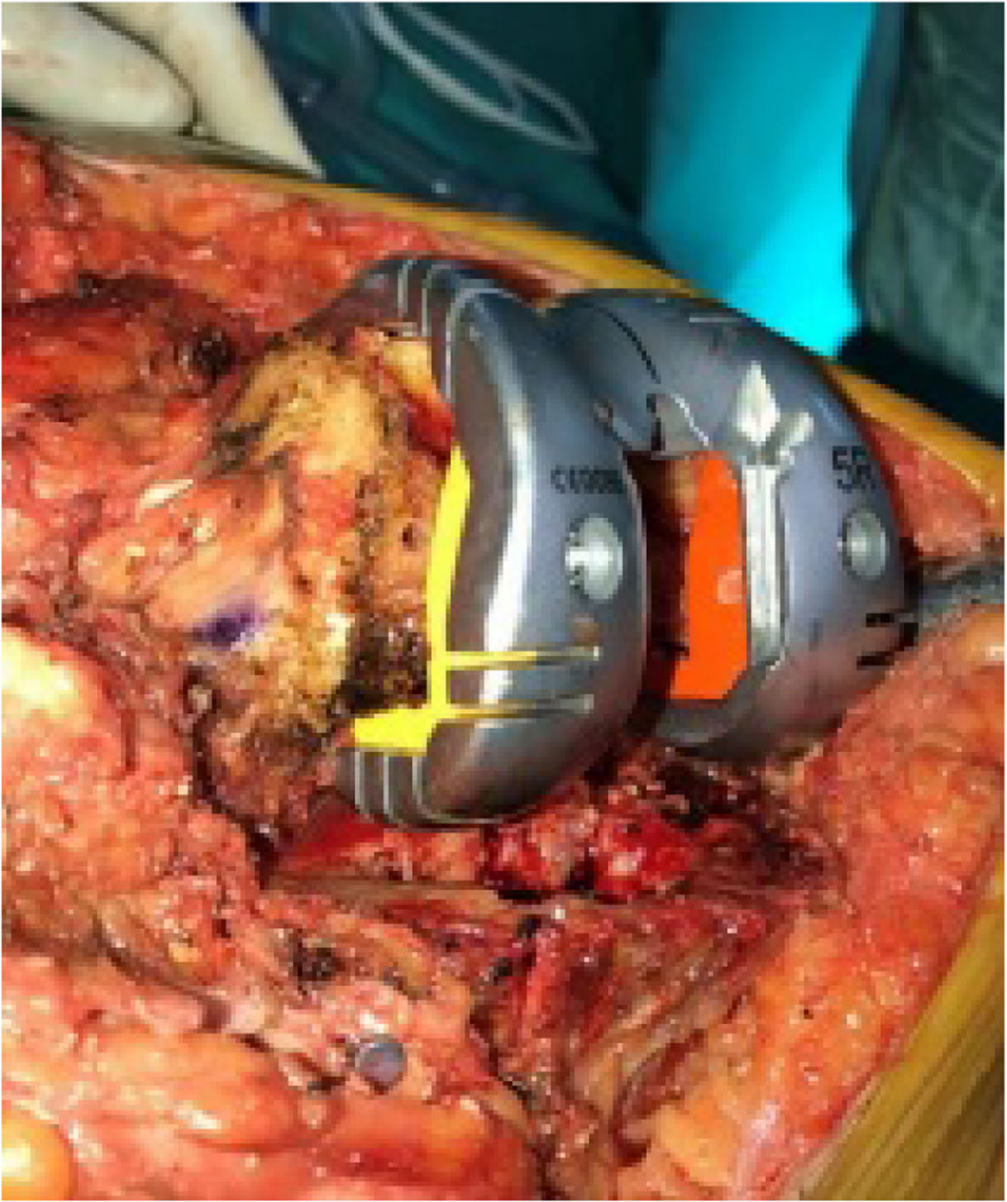
JL and PJL restoration with trial prothesis using augments

**Fig. 5:**
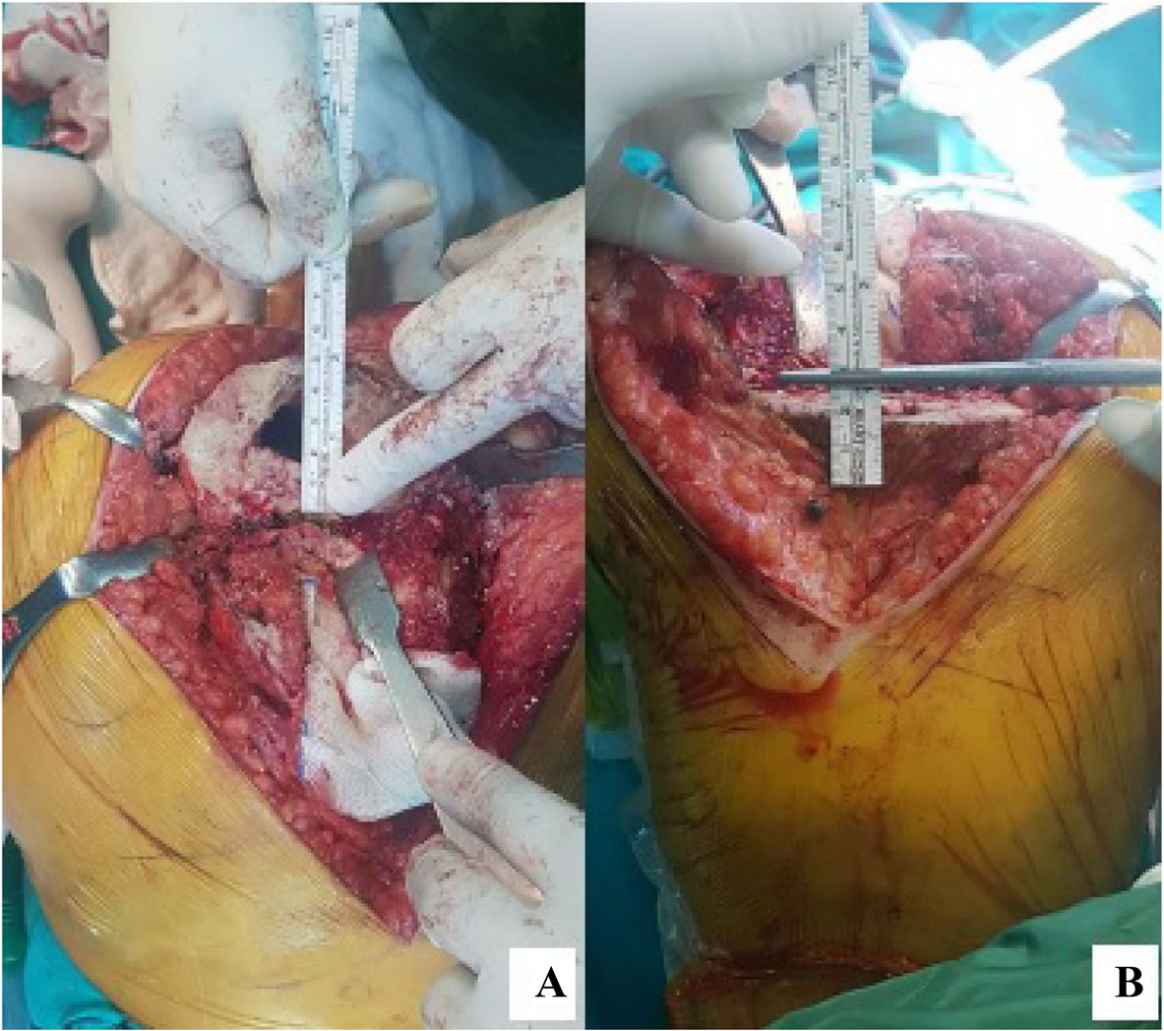
**a.** Measuring the anterior-posterior length of the tibia. **b.** JL restoration according to TT

Distal femoral augments were placed by measuring the distance between the trial prosthesis and the recipient bone tissue. Then, ME-JL was obtained by multiplying FW by 0.34. To obtain the LE-JL, a 0.28 coefficient was multiplied by FW. These values were also compared with AT-JL (Tables 1 and 2) (Fig. 2). In order to reconstruct the PCO, that is, PJL, Servien et al (9) measured the distance from ME and LE to the posterior articular cartilage and described the ratio of this distance to FW. In group 1, ME-PJL was calculated to be 34% of FW and LE-PJL was calculated to be 29% of FW, and PJL was reconstructed according to these calculated values (Tables 1 and 2) (Fig. 3). Distal posterior femoral augments were used to provide calculated distances for the formation of PJL (Fig. 4). JL can also be restored by making calculations distal to the joint. Servien et al (9) suggested that JL should be 50% of the anterior posterior width of the tibia (Fig. 5). In addition, 27% of FW was calculated and TT-JL distance was evaluated on the basis of this value. The size of the tibial prosthesis was determined with regard to the lateral condyle. If necessary, offset stems and metal wedges were added to provide the specified JL, and the rotation of the tibial prosthesis was placed in the posterior cortex of the tibia with an external rotation of 10° (8). In addition, the rotation of the tibial prosthesis was also controlled by TT and the anteromedial cortex of tibia. During the trial, attention was paid to the fact that the knee joint was not in recurvatum while it was in full extension and there was no extension loss. In the flexion arch, any flexion instability (condylar lift-off) was tested and the balance of the joint and the tension of the quadriceps tendon in the flexion position were evaluated manually. Capsule was closed from the patella proximally with a towel clamp from one point and patellofemoral motion was tested. Range of motion and the presence of instability were evaluated especially in flexion between 30°–90°. Lateral parapatellar release was not performed in any knee. It is understood from here that the prostheses were placed in proper rotation. Following the removal of the trial prostheses, the same size prostheses, wedges and stems were implanted. The thickness and length of the prosthetic stems were based on the shortest and widest stems to achieve stability. Prostheses were placed using bone cement with gentamycin (DePuy CMW 1, A B 27 Labios AF cement 1G) given and the stems without cement. Afterwards, a hemovac drain was placed and closure was done accordingly.

**Table 2:** Difference between JL that should be according to FW and JL measured on postoperative X-rays

	**Group 1 (16)**	**Group 2 (16)**	
**AS**^**a**^	**Mean ± SD**^**b**^	**Mean ± SD**	**p**
**AT**^c^**-JL**^**d**^ **(FW**^**e**^**×0.53)**	0.75 ± 0.77	3,06 ± 1,65	< 0.001
**TT**^**f**^**-JL**^**d**^ **(FW**^**e**^**×0.27)**	1.00 ± 0.73	2,88 ± 1,15	< 0.001
**TT**^**f**^**-PJL**^**g**^**(FW**^**e**^**×0.27)**	1.50 ± 0.63	3,19 ± 1,33	< 0.001
**IS**^**h**^ **Index**	1.08 ± 0.16	0.96 ± 0.13	0.026

^a^after surgery,^b^standart deviation. ^c^adductor tubercle, ^d^joint line, ^e^femoral width, ^f^tibial tubercle, ^g^posterior JL,^h^Insall Salvati

### Typical case in group 2

JL was established in group 2 according to the traditional distances of medial epicondyle, lateral epicondyle, fibular head, i.e., “one or two finger(s) proximal to the fibular head or 1.5 to 2 cm and 2.5 to 3 cm to distal the lateral epicondyle and medial epicondyle, respectively”. Ratios method was not used in group 2. Patients were examined regularly in the outpatient clinics. Radiographs were assessed against the criteria developed by Iacono et al [13, 14] and measurements were recorded at each examination.

SPSS 15.0 for Windows (Chicago, IL, USA) was used for statistical analysis. Descriptive statistics were presented as numbers or percentages; categorical variables were expressed as mean, standard deviation, and minimum, maximum and median were used for numeric variables. Student-*t* test was used for between-group comparisons between normally-distributed numeric variables. The Mann Whitney U test was used when the variables were not normally distributed. Chi-square analysis was used for ratio comparison. Correlation between numeric variables was assessed by using Pearson correlation analysis if they were normally distributed and with Spearman Correlation Analysis if they were not. Alfa’s meaningful value was set at 0.05.

## Results

### Before rTKA

Mean age was greater in group 1 than in group 2 [71.0 ± 7.0 (52–81), 65.2 ± 8.1 (52–81), respectively (*p* = 0.038)]. Mean follow-up time was 23.0 ± 9.6 (13–45) months in group 1 and 20.4 ± 6.1 (12–33) months in group 2 (*p* = 0.610). There were 10 right and 6 left knees in group 1 whereas there were 7 right and 9 left knees in group 2 (*p* = 0.228).

There was no difference between 2 groups in terms of flexion, extension and range of motion (ROM) (*p* = 1.000, *p* = 0.619, *p* = 0.773, respectively). There was no difference either between the two groups in KSS Knee and function and WOMAC scores (*p* = 0.462, *p* = 0.657, *p* = 0.323, respectively). The sub-item results of all Short Form 36 (SF-36), except Mental Health scores, were similar. Scores on the Mental Health scale in group 1 were lower before the operation in group 1 (*p* = 0.015). As 2 patients died of non-orthopedic reasons in group 1, 16 knees were radiologically evaluated whereas 14 knees were functionally assessed.

### After rTKA

Improvements in flexion, extension and ROM after surgery were similar (*p* = 0.232, *p* = 0.211, *p* = 0.403, respectively). Mean flexion was higher in group 1 than in group 2 at last evaluation (*p* = 0.046). Mean extension and ROM in both groups were similar and not statistically significant (*p* = 0.064, *p* = 0.069). KSS Knee and Function scores were both significantly higher in group 1 (*p* = 0.031, *p* = 0.036) but the WOMAC scores showed no significant difference (*p* = 0.080). Nonetheless, improvements in KSS Knee and Function and WOMAC were greater in group 1 (*p* = 0.007, *p* = 0.017, *p* = 0.019, respectively).

Improvements in SF-36 scores and its sub-item scores were identical in both groups (*p* = 0.058, 0.556, 0.243, 0.227, 0.599, 0.148, 0.584, 0.302, respectively). All the scores of SF-36 evaluation were the same in the two groups. Regarding the results before surgery, the scores on the ‘Physical function, Physical role functioning, Bodily pain, General health perceptions’ on SF-36 scale were improved in both groups after surgery (*p* = 0.001/0.002, *p* = 0.002/0.001, *p* = 0.001/0.001, *p* = 0.001/0.001). But, ‘Vitality’ and ‘Social role function’ were only improved in group 1 after surgery (*p* = 0.011/0.052, *p* = 0.001/0.055). ‘Emotional role function’ and ‘Mental health’ did not improve in both groups (*p* = 0.067/0.344, *p* = 0.218/0.817).

There was no knee with AORI type 3 bone defect. Two knees in group 1 received tibial tubercle osteotomy and one of them was managed with Achilles tendon allograft with bone because of nonunion of tibial tubercle and extensor mechanism dysfunction afterward. Partial patellar tendon avulsion was repaired in 2 knees and one of them was managed with Achilles tendon allograft with bone. Two knees were debrided and irrigated with an exchange of inserts because of persistent drainage of wound.

The differences between the JL values ​​using the FW according to AT and TT and the JL values ​​that is according to the anteroposterior tibia length and the postoperative JL values ​​were all less in group 1, indicating that JL was restored better. Besides, the postoperative mean IS index was more than that of group 2 (Table 2).

In knees in which AT-JL and TT-JL were established within acceptable limits, KSS Knee and Function scores were higher after surgery (*p* = 0.007, *p* = 0.011) and (*p* = 0.029, *p* = 0.03), respectively and improvement in the mean scores of KSS Knee and Function scores after surgery was also better [(*p* = 0.019, *p* = 0.042) and (*p* = 0.005, *p* = 0.003), respectively]. When ATJL was established within acceptable limits, WOMAC scores were better (*p* = 0.026) (Table 3). Flexion arc, ROM, KSS Knee and Function and WOMAC scores and the improvements of KSS Knee and Function scores after surgery were worse in rTKAs with JL elevation > 4 mm (*p* = 0.010, *p* = 0.014, *p* = 0.004, *p* = 0.003, *p* = 0.30, *p* = 0.002 *p* = 0.03, respectively) (Table 4). ‘Physical function’ and ‘Social function’ were worse in rTKAs with JL elevation > 4 mm (*p* = 0.008 *p* = 0,023, respectively). Improvement in ‘Physical function’, ‘Social function’ and ‘Bodily pain’ was less in rTKAs with JL elevation > 4 mm (*p* = 0.022, *p* = 0.032, *p* = 0.049, respectively).

**Table 3:** Correlations and Differences

	**IS**^**a**^ **Index**		**ATJL**^**b**^		**TTJL**^**c**^	
	**rho**	**p**	**rho**	**p**	**rho**	**p**
**AS**^d^						
**Flexion**	0.368	0.045	−0.306	0.100	−0.087	0.646
**Extension**	−0.181	0.338	0.352	0.057	0.171	0.366
**ROM**^e^	0.256	0.172	−0.318	0.086	−0.113	0.553
**KSS**^f^ **Knee**	0.402	0.028	−0.481	0.007	−0.399	0.029
**KSS Function**	0.359	0.051	−0.460	0.011	−0.448	0.013
**WOMAC**^**g**^	− 0253	0.177	0.407	0.026	−0.087	0.646
**Difference between AS-BS**^h^						
**Flexion**	−0.073	0.702	0.167	0.377	0.193	0.306
**Extension**	0.265	0.158	−0.391	0.033	−0.317	0.088
**ROM**	−0.001	0.997	0.170	0.370	0.205	0.278
**KSS Knee**	−0.298	0.109	0.427	0.019	0.502	0.005
**KSS Function**	−0.220	0.244	0.374	0.042	0.518	0.003
**WOMAC**	0.204	0.280	−0.247	0.187	0.193	0.306

^a^Insall Salvati, ^b^Adductor tubercle JL, ^c^Tibial tubercle JL, ^d^After surgery, ^e^Range of Motion, ^f^ Knee Society Score, ^g^Western Ontario and McMaster Universities Osteoarthritis Index, ^h^Before surgery

**Table 4:** Joint Line Evaluations

Joint Line Elevation			
	**≤ 4 mm**	**> 4 mm**	**p**
	**Mean ± SD**	**Mean ± SD**	
**AS**^**a**^			
**Flexion**	104.3 ± 11.6	90.7 ± 11.0	0.010
**Extension**	2.17 ± 4.73	3.6 ± 3.8	0.166
**ROM**^**b**^	101.3 ± 12.4	87.1 ± 13.2	0.014
**KSS**^**c**^ **Knee**	77.8 ± 15.5	43.7 ± 26.5	0.004
**KSS Function**	77.2 ± 17.5	47.9 ± 22.7	0.003
**WOMAC**^**d**^	12.8 ± 10.5	29.7 ± 19.4	0.016
**Difference between AS and BS**^**e**^			
**Flexion**	25.7 ± 18.5	24.3 ± 18.8	0.866
**Extension**	9.1 ± 7.0	7.9 ± 2.7	0.271
**ROM**	33.9 ± 23.3	33.6 ± 19.1	0.972
**KSS Knee**	67.7 ± 18.6	38.6 ± 23.6	0.002
**KSS Function**	56.7 ± 19.5	33.6 ± 23.0	0.013
**WOMAC**	70.2 ± 15.6	53.1 ± 22.2	0.030

^a^After surgery, ^b^Range of Motion, ^c^Knee Society Score, ^d^Western Ontario and McMaster Universities Osteoarthritis Index, ^e^Before surgery

The mean size of insert was 12.7 ± 2.0 in group 1 and 12.9 ± 0.9 in group 2. In group 2, metal block augmentation was used under the tibial component in 2 patients in group 1 and 10 patients in group 2 (*x*^2^; *p* = 0.011).

## Discussion

Near-anatomic JL establishment is very important in knee arthroplasty [12, 16, 17]. In the current study, JL reestablishment according to the ratios of AT and TT to FW, described in recent years, was found to provide better clinical and radiological results than the traditional distance methods.

One of the results of JL elevation is a decrement in flexion arc [10, 18, 19]. All rTKAs in group 1 had JLs with **≤**4 mm elevation and flexion arc and ROM were better in all knees in group 1. Moreover, KSS Knee and Function scores and WOMAC scores were also better in group 1. We think that JL elevation > 4 mm worsened the clinical results after rTKA in view of the results of our study and JL restored according to ratios of femoral width with adductor tubercle and tibial tubercle yielded higher scores of KSS Knee and improved KSS Knee and Function.

Many surgeons believe that JL should be 2 cm or one to two finger(s) wide proximal to the fibular head in rTKA. Unfortunately, there is no standard available and it is not always possible to be within the threshold of 4 mm. Servien et al [9] found the mean distance from fibular head and JLwas 14 (4.1–22.3) mm. Since Figgie method for JL restoration has been said to be misleading [16] because of that reason, a landmark located in the distal femur and the ratios with regard to FW have been offered instead of the proximal tibia [12]. We think the reason why JL restoration was worse in group 2 might be that the reference landmark in group 2, i.e., the fibular head, is distal to the knee joint. So, we suggest the fibular head should not be used as a landmark for restoring the JL when performing rTKA.

TT ratio (27% of FW or 50% of AP tibial length) must be used instead of fibular head distances if one intends to use a landmark in proximal tibia [9]. Mean TT-JL distance was 1.0 ± 0.73 (0–2) mm in group 1 and 2.28 ± 1.15 (1–5) mm in group 2. We strongly believe that JL restoration from the tibial side was more physiologic in group 1. In view of our results, we think that TT (27% of FW or 50% of AP tibial length) can be used as a landmark for restoring the JL in rTKA instead of some traditional distances from the fibular head.

Referencing either the radiographs of the revised knee before index operation or the radiographs of contralateral knee that should be free of knee replacement is very difficult and not practical as it is easily appreciated [7, 17, 19, 20]. Because of those reasons, we suggest reestablishment of JL in rTKA by using ratios of FW concerning landmarks such as AT and TT.

The relation between JL and patella height has been studied in 74 septic and aseptic rTKAs involving 70 patients and JL level could be maintained with distal femoral augments but, patellar height could not have been improved, especially in septic revision due to obvious patellar tendon contractures and authors concluded that they did not find any relationship between JL level and patella height [21]. In our study, IS index was higher in group 1. After the exclusion of 2 patients with tibial tubercle avulsion and patellar tendon rupture, no difference remained between the two groups. Considering this, our result was similar to the above-mentioned study.

A fluoroscopic study found that posterior JL (PJL) was found to be related to the stability of knee in flexion and ROM [22]. Moreover, a study involving 107 rTKA by Clement et al demonstrated that PJL was an independent variable affecting functional results after surgery [23]. In the current study, we restored the posterior JL with the ratios of FW and ME and LE and 1/3 posterior to the trans-epicondylar axis defined by Servien et al [9] and we think that, since we reestablished the posterior JL anatomically close to normal, the flexion arcs in group 1 were better after surgery.

In our study, the distal femoral augments were thick enough to restore the JL according to the ‘AT-RM’ in group 1. Another study involving 107 rTKA (99 patients) used an insert of a mean size of 17.2 ± 3.85 and thicker distal femoral augments in patients with deficient stock of distal femoral bone [17]. In our study, we used the insert of a mean size of 12.7 ± 2.0 in group 1 and 12.9 ± 0.9 in group 2. In group 2, metal block augments were used under the tibial component in 2 (two) patients in group 1 and 10 (ten) patients in group 2 (*p* = 0.011). This explained absence of difference in insert thickness between two groups.

In our study, 6 patients had anterior knee pain and the patellar tendon rupture of one patient was repaired with an allograft of Achilles tendon with bone in group 1 (I/S: 1.32). Five patients in group 2 had elevated JL. Among the 6 patients, only 1 had an I/S index of 0.8 and the other 5 had the index between 0.8–0.92. Anterior knee pain was ascribed to JL elevation. The authors concluded that patella baja could be corrected by osteotomy of the tibial tuberosity so that a proximalisation of one to two centimeters could be achieved [24]. In our study, anterior knee pain was not the aim of primary research. But we think that the anterior knee pain in the above-mentioned patients in our study might have been caused by patella baja.

Non-union after tibial tubercle osteotomy in one patient in group 1 was repaired with Achilles tendon allograft with bone. The functional result of this patient was poor than the other patients in group 1. The other patients receiving tibial tubercle osteotomy was free of non-union complications. Barrack et al [25] reported that they used tibial tubercle osteotomy in 15 patients out of 123 rTKAs in their cohort and found that functional results of these 15 patients were poor.

Our study has both advantages and disadvantages. Although the mean age of patients in group 1 was older, we had higher functional and clinical results in the patients in group 1 after surgery. ‘Mental health’ of SF-36 was also worse in group 1. But we think that JL restored to near-to-normal anatomy in all knees in group 1 yielded more favorable clinical and radiological results and that eliminated this disadvantage. The other point is that we had a relatively small number of patients but, we collected the data prospectively and we had no patient who was lost. This is the main advantage of our study.

## Conclusions

Our results showed that restoration of JL to near normal in rTKA can be achieved with ‘AT-RM’ and this method was superior to the traditional ‘distances method’. Besides, restoration of PJL, also known as posterior condylar offset, had a direct positive effect on flexion arc. Since this ratio method eliminates the necessity of preoperative X-rays of index TKA, we suggest to use it in JL reestablishment in rTKA both in coronal and sagittal planes, which can achieve a good functional outcome and higher patient satisfaction. If one intends to use a landmark distal to the knee joint, ratios of TT related to FW and AP tibial length can be used to restore the JL in the coronal plane. TT ratios can also be used for checking the JL measured from the femoral side.

## Data Availability

Not applicable.

## Abbreviations

AL-DM: Anatomic Landmark Distance Method
JL: Joint Line
TKA: Total Knee Arthroplasty
AT-RM: Adductor Tubercle Ratios Method
KSS: Knee Society Score
WOMAC: Western Ontario and McMaster Universities Osteoarthritis Index
SF-36: Short Form-36
AT: Adductor Tubercle
TT: Tibial Tubercle
LE: Lateral Epicondyle
ME: Medial Epicondyle
FW: Femoral Width
PJL: Posterior Joint Line
TEA: Transepicondylar Axis
AORI: Anderson Orthopedic Research Institute
PCO: Posterior Condylar Offset
AP: Anterior - Posterior
ROM: Range of Motion
